# Leveraging Two Kinect Sensors for Accurate Full-Body Motion Capture

**DOI:** 10.3390/s150924297

**Published:** 2015-09-22

**Authors:** Zhiquan Gao, Yao Yu, Yu Zhou, Sidan Du

**Affiliations:** School of Electronic Science and Engineering, Nanjing University, Nanjing 210046, China; E-Mail: gaozq1992@163.com

**Keywords:** motion capture, pose estimation, temporal constraint, Kinect sensors

## Abstract

Accurate motion capture plays an important role in sports analysis, the medical field and virtual reality. Current methods for motion capture often suffer from occlusions, which limits the accuracy of their pose estimation. In this paper, we propose a complete system to measure the pose parameters of the human body accurately. Different from previous monocular depth camera systems, we leverage two Kinect sensors to acquire more information about human movements, which ensures that we can still get an accurate estimation even when significant occlusion occurs. Because human motion is temporally constant, we adopt a learning analysis to mine the temporal information across the posture variations. Using this information, we estimate human pose parameters accurately, regardless of rapid movement. Our experimental results show that our system can perform an accurate pose estimation of the human body with the constraint of information from the temporal domain.

## 1. Introduction

The ability to accurately recover pose parameters plays an important role in sports analysis, the medical field and virtual reality. In the past few decades, great development has been achieved in the technology of motion capture [1]. One common solution is to use marker-based motion capture systems (e.g., Vicon Systems [2]), inertial motion capture (e.g., Xsens [3]) and magnetic motion capture (e.g., Ascension [4]). On the whole, these solutions can accurately and reliably capture full-body kinematic motion parameters, but they are often cumbersome, expensive and intrusive. As a replacement, marker-less multi-view systems [5,6,7,8] have become more and more popular in the motion capture field to acquire poses. However, these solutions always take too much space and are difficult to set up.

With an in-depth study on the accuracy of the Microsoft Kinect sensors [9], monocular depth camera systems [10,11] have been a better choice for motion capture. Methods for pose estimation with monocular depth camera systems can be divided into three parts: example-based approaches, bottom-up approaches and top-down approaches. Shotton *et al.* [12] transformed the pose estimation problem into a simpler per-pixel classification problem by designing an intermediate representation in terms of body parts. This solution belongs to a bottom-up method, and poses can be estimated without any initialization. However, this solution needs a large number of training sets and always outputs bad results when significant occlusion occurs. Ye *et al.* [10] proposed an example-based technology in computer vision to estimate body pose configuration from a single depth map. This solution matches the input depth map with a set of pre-captured motion exemplars to generate a body configuration estimation, as well as semantic labeling of the input point cloud. However, motion capture may fail because of large skinning deformation, and many exemplars should be matched when searching for the best matched one. Top-down approaches are the most common methods in motion capture with monocular depth camera systems. Grest *et al.* [13] applied iterative closest point (ICP) techniques to register an articulated mesh model with monocular depth data along with silhouette correspondences between the hypothesized and observed data. However, the ICP algorithm is often sensitive to initial poses and prone to local minima, thereby failing to track 3D human poses from noisy depth data obtained from a single camera. Furthermore, Wei *et al.* [14] formulated the registration problem in a maximum *a posteriori* (MAP) framework and iteratively updated 3D skeletal poses with monocular depth data via a linear system. Because this solution often output poor results for roll motion, Xu *et al.* [15] adapted the SCAPEmodel [16] for the pose estimation process and acquired a more accurate model with large-scale motion.

Overall, current top-down approaches for pose estimation with monocular depth camera systems are wholly parametric methods, which are prone to local minima, as significant occlusion occurs. Since the problem of local minima still is not solved, what we can do is to avoid it as much as possible by collecting more movement information. Therefore, in this paper, we propose a system using two Kinect sensors to collect motion data, including RGB images and depth images, from two different viewpoints at the same time. In addition, we apply a temporal constraint to improve the accuracy of pose estimation. For pose estimation of the current frame, we just use the former estimated poses instead of the depth and RGB images to predict the pose of the next frame, which will be used to ensure that our estimation of the current frame is consistent with both the estimated last pose and the predicted next pose at the same time. The prediction of the next pose is seen as a true and known result of the next frame during the process of optimization for the current frame. For Shen *et al.* [17,18,19], the temporal constraint uses the predicted corrected poses to optimize the offset of all skeletons and the prediction follows the change of skeleton offset. We acquire an accurate SCAPE model leveraging non-rigid deformation of the template mesh with the help of Kinect Fusion [20]. Then, we combine pose detection with pose tracking as a multi-layer filter to estimate accurate pose parameters automatically from two Kinect sensors at the same time. We prove the importance of leveraging two Kinect sensors by comparing estimated poses from monocular depth camera systems and a two-Kinect sensor system. Furthermore, we demonstrate the necessity of the temporal term by comparing the results under the constraint of the temporal term against the output poses without the temporal term. Afterward, we implement a quadratic and a linear model, respectively, for our temporal term and compare the estimated poses of these two different methods.

In summary, our contributions are: (1) a two-Kinect sensor system instead of monocular depth camera systems for motion capture in the noisy and low-resolution conditions, which makes our pose estimation still accurate when significant occlusion occurs; the combination of pose detection, pose tracking and failure detection makes our human pose estimation accurate and automatic; (2) the application of the temporal constraint term with the quadratic function to track poses, which makes our estimation achieve higher accuracy; (3) using skeletons from Kinect sensors to calibrate the position and orientation of the two Kinect sensors, which makes our depth camera calibration easy, but effective.

## 2. Overview

**Figure 1:**
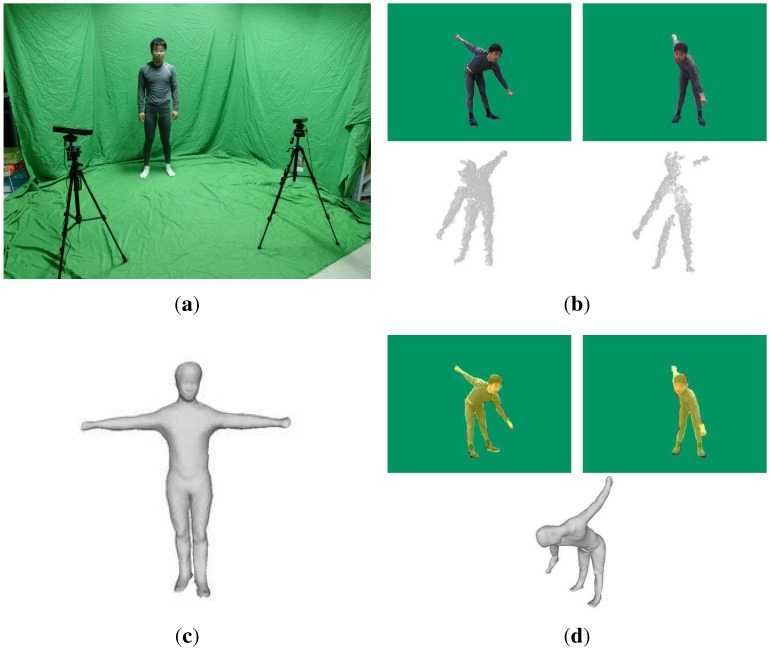
(**a**) Setup of our system; (**b**) data acquisition: RGB image (top) and depth data (bottom) from two Kinect sensors; (**c**) template SCAPEmodel; (**d**) reconstructed SCAPE model (top) and projection (bottom); the yellow shadows are the projections of our reconstructed model.

In this paper, we present a system for measuring the motion parameters of the human body accurately and automatically. At first, we get two video sequences of people in diverse poses captured by the system with two Kinect sensors (Figure 1a), which involves both depth and RGB images (Figure 1b). Afterward, we reconstruct the template SCAPE model of the body shape of the subject (Figure 1c). Then, we recover the pose for each frame in these sequences following a fully-automatic multi-layer framework. The multi-layer framework consists of a pose-detection module, a pose-tracking module and a failure-detection module. For pose detection, we transform this problem into a simpler per-pixel classification problem by designing an intermediate representation in terms of the body, which is similar to [12]. In contrast to [12], our synthetic training database consists of SCAPE models instead of point cloud models. Another difference between our method and [12] is that we have two input depth images from two Kinect sensors, while only one in [12]. Making full use of the two input depth images, we align the depth data from the two cameras to obtain a point cloud using the rigid transformations obtained from the calibration step. To cope with the instability of the aligned point cloud provided by the Kinect and calibration error, we firstly discard some error points according to the angles between the normal of the points and z-directions of the two Kinect, where the angles are set to be less than $90^{\circ }$. Then, a temporal denoising method [21] is utilized to smooth and refine our aligned point cloud, which makes our pose detection more robust and more accurate, even though occlusion occurs. For the pose tracking, we further extend the method proposed by Xu [15]. We leverage a two-Kinect sensor system to acquire human motion data instead of a monocular sensor, and we adopt the temporal constraint, which means we make full use of the correlation of human motion among continuous frames temporally. Our pose tracking provides highly accurate estimation for each frame (Figure 1d), while pose detection provides coarse pose estimation for automatic initialization/reset in the 3D pose tracking process.

## 3. Motion Capture

In this section, we introduce our motion capture module based on the SCAPE model. Firstly, we introduce a method similar to [22] to capture the human full-body shape, which is necessary for our pose tracking, as we can get a quite accurate shape model in this easy way. Afterward, we represent a new method for point cloud registration from the two Kinect sensors. Then, we introduce our pose estimation using data from two Kinect sensors with the proposed temporal constraint term. Because our pose-tracking module can fail sometimes, as it may fall into local minima, we use a failure-detection module to detect failed pose tracking, finally, so that we call the pose-detection module to provide coarse pose estimation for initialization. In the following parts of our work, we name our two Kinect sensors the left and right sensor for brevity to distinguish them.

### 3.1. Full-Body Shape Modeling

We use SCAPE to model the human full-body shape, as the SCAPE model can capture human bodies with complex non-rigid deformations, especially large skinning deformation, induced by both pose and shape variation. The SCAPE model is a parametric method of modeling human bodies and is learned from a database of several hundred laser scans. The database that we used to train the SCAPE model is from [23], and our SCAPE model consists of 12,500 points and 25,000 triangles. We approximate the human body geometry with 16 rigid parts as a tree-structured articulated skeleton and use 42 degrees of freedom (DoF) to describe people’s different poses. Therefore, we will represent the pose parameters that are estimated for the human body as $q\in R^{42}$, where the first six degrees of freedom represent the absolute root position and orientation of the right sensor, the second six degrees of freedom represent the left sensor and the other degrees of freedom represent the relative joint rotations. These joints are neck (three DoF), upper back (one DoF), waist (two DoF), left and right shoulder (three DoF), elbow (two DoF), wrist (one DoF), hip (three DoF), knee (two DoF) and ankle (one DoF).

**Figure 2:**
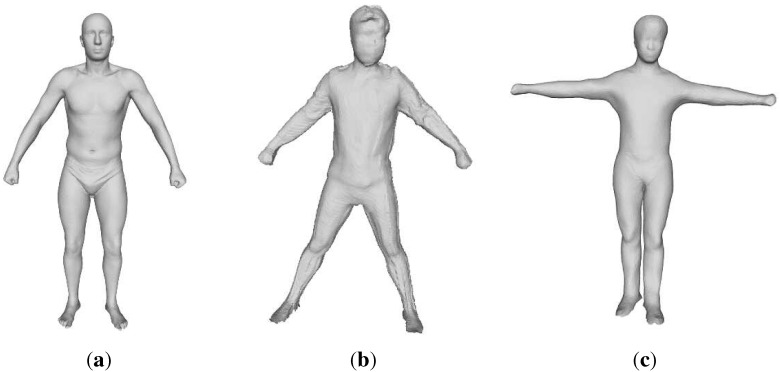
(**a**) SCAPE model; (**b**) scanned point cloud; (**c**) template SCAPE model.

To acquire a template SCAPE model, we use a Kinect sensor to scan a person by Kinect Fusion and get the point cloud of the whole body of a person (Figure 2b), which shows the person’s relatively accurate shape. Then, it is transformed to a template SCAPE model, using non-rigid deformation of the template mesh to fit the point cloud from the Kinect sensor to a template SCAPE model. Firstly, we train a model from our trained SCAPE model from [24], called the SCAPE model in the following sections, the pose of which is similar to the pose of our scanned person, called the scanned point cloud. Then, using the joints from the trained SCAPE model and that from the Kinect as correspondence points, the rotation matrix Ro and translation matrix Tr between these correspondence points are calculated. Afterward, we iteratively transform points that surround these markers by a smoothness term to fit the SCAPE model to the scanned point cloud. After iteration is terminated, the missing data in the scans can be filled using the template, and we smooth the transformed SCAPE model using the uniform Laplacian coordinates [25]. A full-body SCAPE model of the person is completed and transformed into a template pose (Figure 2c) using the inverse linear blending skinning (LBS) model. We weigh every vertex in these models of the template pose according to the z-direction error property of the Kinect and whether the vertex is in front of the sensor when the model it belongs to is in its original pose.

### 3.2. Point Cloud Registration

Reconstructing 3D body poses using multiple depth cameras requires computing the relative positions and orientations of all depth cameras.

**Figure 3:**
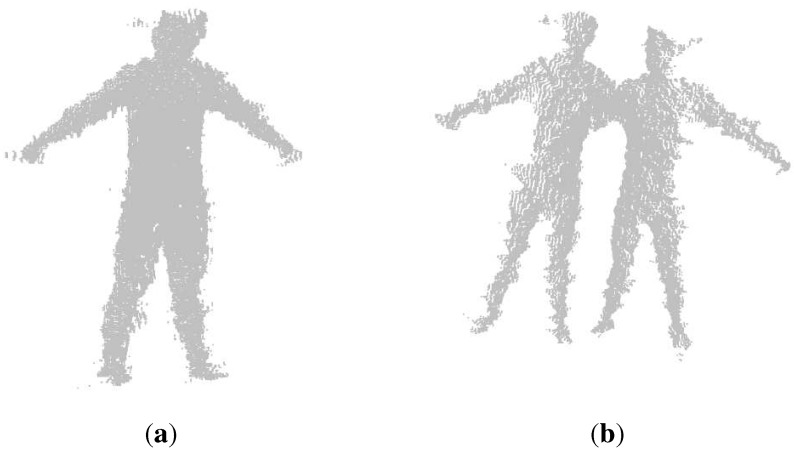
Registration results: (**a**) registration results using our calibration method; (**b**) registration results using stereo calibration in the calibration toolbox of MATLAB.

Berger in [26] re-investigated the magnitude of interferences in a motion capturing setup with multiple Kinects. For depth camera calibration, he proposed to use a checkerboard consisting of white diffuse and mirror patches or a point light source with a mirror disc attached to create a depth checkerboard pattern. This method is inconvenient, as a special checkerboard is needed, and it may not work very well on the edges of these white diffuse and mirror patches, due to absence of some IR dots (from the projected pattern) within a point neighborhood. Auvinet in [27] used multiple Kinects for body volume reconstruction for gait analysis. He calibrated the external parameters of these Kinects based on several plane intersections, which were obtained by detecting the edges and corners of a large planar rectangle. This method is convenient and simple, but may suffer from incorrect detection of the large planar rectangle due to the noise of the Kinects. Another simple method is to use the external parameters of RGB cameras to replace that of the depth sensors. However, because there is an external rotation and translation matrix between depth and RGB cameras, utilizing the resulting external matrix between RGB cameras to calibrate the depth cameras will lead to an inaccurate result. Therefore, we adopt a new method of utilizing skeletons from the Kinect for calibration instead of calibrating via RGB cameras. Firstly, we choose frames from our sequences in which human poses are easy to capture by both of the two Kinect sensors. Then, the joints of the skeletons from the Kinect in these frames are registered, and the calibration result is further optimized, because it may not be accurate due to the error of the skeletons from the Kinect sensor. We derive the calibration matrix by registering the right depth image to the left one with ICP. Figure 3 shows the comparison of our calibration result with the one derived using RGB cameras, which shows that our calibration obtains a more accurate result for the relative positions and orientations of the depth cameras of these two Kinect sensors.

### 3.3. Pose Tracking

The core of our pose-tracking algorithm is to formulate the registration problem in an MAP [28] framework. We solve the pose parameters, $q\in R^{42}$ and register the 3D articulated human body model with depth and RGB cues via linear system solvers. Let $C_{i}$ be the input data at the current frame i, which consists of a depth image ($D_{i}$), an RGB image ($R_{i}$) and a binary silhouette image ($S_{i}$). In addition, the depth maps we get from the Kinect sensors are depth values on the 2D plane. We project them onto the world coordinate system with calibration parameters and get the depth images (*D*) for input data (*C*). Binary silhouette images (S) in the input data (*C*) are extracted from the depth images instead of silhouette images from the Kinect sensors, as there are not only the kinematic persons’, but also the surroundings’ silhouette contours in these images. We denote the sequence of *m* previously reconstructed poses as $Q_{m}^{i}=\left[q_{i-1},\ldots ,q_{i-m}\right]$, and we assume the reconstructed pose of the (i+1)-th frame as $q_{i+1}$. Therefore, we denote the *m* constructed poses and the (i+1)-th assumed pose together as $Q_{m+1}^{i}$. The most likely pose $q_{i}^{*}$ for the *i*-th frame can be estimated by solving the following MAP estimation problem:

(1) $$q_{i}^{*}=\arg\max_{q_{i}}\underset{Likelihood}{\underset{︸}{Pr(C|q_{i})}}\underset{Prior}{\underset{︸}{Pr(q_{i}|Q_{m+1}^{i})}}$$

In Equation (1), we formulate the likelihood term and still use the SCAPE model for tracking, similar to Xu *et al.* [15]. However, we propose a temporal term for the prior term to utilize the temporal information. Our prior term now is formulated as:

(2) $$Pr\left(q_{i}|Q_{m+1}^{i}\right)=\underset{Previous}{\underset{︸}{Pr(q_{i}|Q_{m}^{i})}}\underset{Temporal}{\underset{︸}{Pr(q_{i}|q_{i+1})}}$$

where the first term $Pr(q_{i}|Q_{m}^{i})$ describes the prior distribution of the current pose $q_{i}$ given the previous reconstructed poses $Q_{m}^{i}$ and the second term $Pr(q_{i}|q_{i+1})$ measures the distribution to the current pose of the next frame. This proposed temporal term ensures that the estimated pose is consistent with both the previous and future poses, which avoids unreasonable large changes in adjacent frames.

#### 3.3.1. Temporal Term

Since bones of a person cannot rotate unlimitedly, the previous term $Pr(q_{i}|Q_{m}^{i})$ can result in bad estimation when the person moves at a fast speed, which means the rotation angle between adjacent frames is large. The term $Pr(q_{i}|Q_{m}^{i})$ is used to ensure $q_{i}-q_{i-1}\approx q_{i-1}-q_{i-2}$, which is based on an assumption that kinematic motion is slow and constant between adjacent frames. However, if the human current motion is not constant with previous poses, this term may lead to a poor estimation in pose change. Therefore, we propose the temporal term, which aims to keep constant among the current pose, previous pose and future pose. We optimize current pose $q_{i}$, making $q_{i}$ a transition between $q_{i-1}$ and $q_{i+1}$, where $q_{i+1}$ is the pose parameter of the next (i+1-th) frame predicted with information from the temporal term. Specifically, we try to ensure $q_{i}-q_{i-1}\approx q_{i+1}-q_{i}$. Therefore, this temporal term can be formulated as:

(3) $$Pr\left(q_{i}|q_{i+1}\right)=\frac{1}{\sqrt{2\pi }\sigma_{t}}\exp\left(\frac{-∥q_{i+1}-2q_{i}+q_{i-1}{∥}^{2}}{2\sigma_{t}^{2}}\right)$$

Therefore, the prior term can be formulated as:

(4) $$Pr\left(q_{i}|Q_{m+1}^{i}\right)=\frac{1}{\sqrt{2\pi }\sigma_{s}}\exp\left(\frac{-∥q_{i}-2q_{i-1}+q_{i-2}{∥}^{2}}{2\sigma_{s}^{2}}\right)\frac{1}{\sqrt{2\pi }\sigma_{t}}\exp\left(\frac{-∥q_{i+1}-2q_{i}+q_{i-1}{∥}^{2}}{2\sigma_{t}^{2}}\right)$$

**Figure 4:**
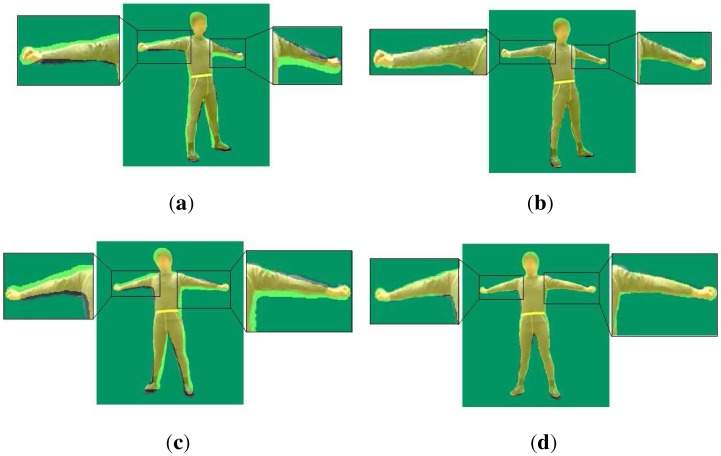
Impact of our temporal term: the yellow shadow on these four pictures is the projection of our model transformed with the estimated pose parameters. (**a**) projection of the reconstructed model for the left camera without our temporal term; (**b**) projection of our reconstructed model for the left camera with our temporal term; (**c**) projection of the reconstructed model for the left camera without our temporal term; (**d**) projection of our reconstructed model for the left camera with our temporal term.

Note that the temporal term forms similarly to the previous term in the MAP formula, which means that we can optimize this term in the same way that we optimized the previous term in Equation (12). When optimizing the temporal term, we should predict the pose $q_{i+1}$ of the next frame ((i+1)-th frame) accurately. The prediction is critical to the resulting accuracy of pose estimation; therefore, it is important to try an accurate way to estimate the pose parameters of the next frame. In practice, we use a high order polynomial to predict the next pose. Denoting the sequence of *m* previously reconstructed poses as $q_{i-1},\ldots ,q_{i-m}$ and supposing we use an *n*-order polynomial function about the time parameter *t* to predict it, we can formulate the next pose parameter $q_{i+1}$ as:

(5) $$q_{i+1}=\sum_{k=0}^{n}\left(a_{k}t^{k}\right)$$

where all $a_{k}\left(k=0,\ldots ,n\right)$ are the coefficients of the polynomial and solved by optimizing equations with the method of machine learning:

(6) $$q_{i-j}=\sum_{k=0}^{n}\left(a_{k}^{\left(i-j\right)}t_{\left(i-j\right)}^{k}\right),for\;j\;=\;1,2,\ldots ,m$$

in which $q_{i-j}$ indicates the reconstructed pose of the (i−j)-th frame, while $a_{k}^{\left(i-j\right)}$ is the coefficients of the *k*-th order of the (i−j)-th frame, and $t_{\left(i-j\right)}$ is the acquisition time of the (i−j)-th frame. We solve Equation (6) by the least squares method and optimize each DoF of $q_{i+1}$. Figure 4 shows the impact of our temporal term. It shows that the projections of arms and legs of the reconstructed model without our temporal constraint wander further from their actual locations than the projections under the temporal constraint. This means that our temporal term makes the pose estimation of the poses of arms and legs more accurate, especially in the case where reconstructed bodies move rapidly.

In practice, if the time interval between the first and last frames for prediction is too large, the predicting function may be over-fitting with too many samples. Therefore, we just use the previous five poses to fit a quadratic polynomial to predict pose $q_{i+1}$ of the next frame. Similarly, a higher-order polynomial function to predict $q_{i+1}$ may lead to over-fitting, and a lower-order polynomial function may lead to under-fitting. We also use a logarithmic and an exponential function for our prediction, but we find that using the quadratic polynomial function obtains the best results among these hypotheses. As shown in Table 1, pose tracking with our temporal term using quadratic prediction always gives appropriate estimations of current poses, which results in an increase of about three percentage points in the average coincidence ration of the effective area, while the pose tracking with our temporal term using linear prediction always gives poorer estimations, even compared to estimations without this term, which results in a decrease of about one percentage point in the average coincidence ration of the effective area. Note that because the human is closer to the right camera and there is little occlusion from the viewpoint of the right camera, all of the coincidence ratios in the depth map of the left camera are slightly less than those of the right camera.

**Table 1:** Comparison of pose estimation with linear and quadratic prediction and without the temporal term. In this table, the coincidence ratio means the proportion of the effective area, where the difference of the depth value between the observed and rendered map is less than 5 cm.

	Left Camera	Right Camera
Coincidence ratio without temporal term	87.51%	88.39%
Coincidence ratio with linear prediction	86.48%	87.43%
Coincidence ratio with quadratic prediction	90.54%	91.68%

#### 3.3.2. Optimization

After adding the temporal term, the MAP estimation problem can be written as:

(7) $$q_{i}^{*}=\arg\max_{q_{i}}Pr_{depth}Pr_{extra}Pr_{silhouette}Pr_{rgb}Pr_{previous}Pr_{temporal}$$

s.t.

(8) $$Pr_{depth}=\frac{1}{\sqrt{2\pi }\sigma_{depth}}\exp\left(\frac{-∥D_{render}\left(x\left(q_{i}\right),q_{i}\right){-D∥}^{2}}{2\sigma_{depth}^{2}}\right)$$

(9) $$Pr_{extra}=\frac{1}{\sqrt{2\pi }\sigma_{extra}}\exp\left(\frac{-∥p\left(q_{i}\right)-p^{*}{∥}^{2}}{2\sigma_{extra}^{2}}\right)$$

(10) $$Pr_{silhouette}=\frac{1}{\sqrt{2\pi }\sigma_{silhouette}}\exp\left(\frac{-∥S_{render}\left(p\left(q_{i}\right)\right){-S∥}^{2}}{2\sigma_{silhouette}^{2}}\right)$$

(11) $$Pr_{rgb}=\frac{1}{\sqrt{2\pi }\sigma_{silhouette}}\exp\left(\frac{-∥p_{rgb}\left(q_{i}\right)-p_{rgb}^{*}{∥}^{2}}{2\sigma_{rgb}^{2}}\right)$$

where x(q) represents 2D pixel coordinates on the depth image, p(q) represents 3D points on the depth map that are rendered from the SCAPE model with the pose parameter q, $D_{render}$ represents the depth map rendered from our SCAPE model, *D* represents the observed depth map, $p^{*}$ represents the points on the observed depth map, $p_{\mathrm{rgb}}^{*}$ represents the points on the current observed depth map and $S_{render}$ represents the points on the silhouette image that are rendered from the SCAPE model. We solve this MAP estimation by minimizing the negative logarithm of the posterior density function and solve the following energy minimization problem:

(12) $$\arg\min_{q_{i}}E_{depth}+E_{extra}+E_{silhouette}+E_{rgb}+E_{previous}+E_{temporal}$$

When we optimize this cost function, we project our SCAPE model to the depth domain to render a hypothesized depth map and divide the depth map into several parts according to the comparison of the rendered depth map and the observed one. We define the region where the difference at the same 2D pixel between the observed and the rendered depth map is no more than 5 cm and both depths are not zero, an overlapping region. Pixels in the overlapping region are taken into the correspondence set for the depth term $E_{depth}$. Pixels in the non-overlapping region, which means the difference at the same 2D pixel between the depth and the observed depth map is over 5 cm, are put into the correspondence set for the extra term $E_{extra}$. For the silhouette term, we adopt the coherent point drift (CPD) algorithm [29] to register the correspondence between the silhouette of the rendered depth map and the observed map instead of searching for the nearest points. For the RGB term, we project our SCAPE model onto the RGB image and find the nearest depth points to be the correspondence of the RGB points. Extending the Lucas–Kanade algorithm [30], we iteratively solve the above non-linear least squares problem via linear system solvers. Performing a first-order Taylor expansion for upper nonlinear expressions, we can obtain the following linear Equations (19)–(24) on $\delta q_{i}$:

(13) $$\frac{1}{\sigma_{depth}}\left(\nabla D_{render}\frac{\partial x}{\partial p}+\frac{\partial D_{render}}{\partial p}\right)\frac{\partial p}{\partial q_{i}}\delta q_{i}=\frac{1}{\sigma_{depth}}\left(D-D_{render}\right)$$

(14) $$\frac{1}{\sigma_{silhouette}}\nabla S_{render}\frac{\partial x}{\partial p}\frac{\partial p}{\partial q_{i}}\delta q_{i}=\frac{1}{\sigma_{silhouette}}\left(S-S_{render}\right)$$

(15) $$\frac{1}{\sigma_{extra}}\frac{\partial p}{\partial q_{i}}\delta q_{i}=\frac{1}{\sigma_{extra}}\left(p^{*}-p\right)$$

(16) $$\frac{1}{\sigma_{rgb}}\frac{\partial p_{rgb}\left(q_{i}\right)}{\partial q_{i}}\delta q_{i}=\frac{1}{\sigma_{rgb}}\left(p_{rgb}^{*}-p\right)$$

(17) $$\frac{1}{\sigma_{s}}\delta q_{i}=\frac{1}{\sigma_{s}}\left(2q_{i-1}-q_{i-2}-q_{i}\right)$$

(18) $$\frac{1}{\sigma_{t}}\delta q_{i}=\frac{1}{\sigma_{t}}\left(q_{i}-\frac{1}{2}\left(q_{i+1}+q_{i-1}\right)\right)$$

where the standard deviations, $\sigma_{depth},\sigma_{extra},\sigma_{silhouette},\sigma_{rgb},\sigma_{s},\sigma_{t}$ are used to control the weights for each term and are experimentally set to 1, 0.08, 100, 0.05, 12.33, 11.5, respectively. In the above equations, image gradients $\nabla D_{render}$ and $\nabla S_{render}$ are evaluated on the rendered depth and silhouette image, respectively. In addition, we do not mosaic the depth images from the left and right camera to get a full cloud point body to estimate human poses. We project our SCAPE model into the depth domain to render a hypothesized depth map and compare the rendered depth map with the observed depth map from the corresponding camera, respectively. In practice, we also optimize the external parameters of these two cameras at each time of iteration using the absolute root position and the orientation of the left and right sensor represented by the first 12 degrees of freedom of the pose parameter *q*, which is a benefit for improving the accuracy of our pose tracking.

### 3.4. Failure Detection

After the pose tracking module, we build a failure detection module to determine when the pose tracking fails and to initiate the pose detection module. Tracking failure is identified by evaluating how well the reconstructed poses match the current input data. We project the rendered model from the reconstructed pose parameters q to the observed depth map from the left camera and the right camera separately. The map is divided into several regions according to the difference between the rendered pixel and observed pixel.

- $Region_{1}$: The pixels belong to both the observed and the rendered map, and the difference of the depth value is less than 5 cm
- $Region_{2}$: The pixels belong to both the observed and the rendered map, but the difference of the depth value is more than 5 cm
- $Region_{3}$: The pixels belong to the rendered depth map, but do not belong to the observed depth map.
- $Region_{4}$: The pixels belong to the observed depth map, but do not belong to the rendered depth.

We call $Region_{1}$ the effective area, and we calculate the proportion of $Region_{1}$ to judge if the current reconstructed pose is effective. To be specific, the system automatically switches to the pose detection if one of the following two conditions is satisfied:

- The proportion of the effective area $Region_{1}$ is less than a threshold, which is experimentally set to 15%.
- Any term of the reconstructed pose parameters comes to an unreasonable result. In other words, an unreasonable result means that the rotation direction or angle comes to an impossible result, for example the rotation angle of the arm is less than 0.

## 4. Results and Discussion

**Figure 5:**
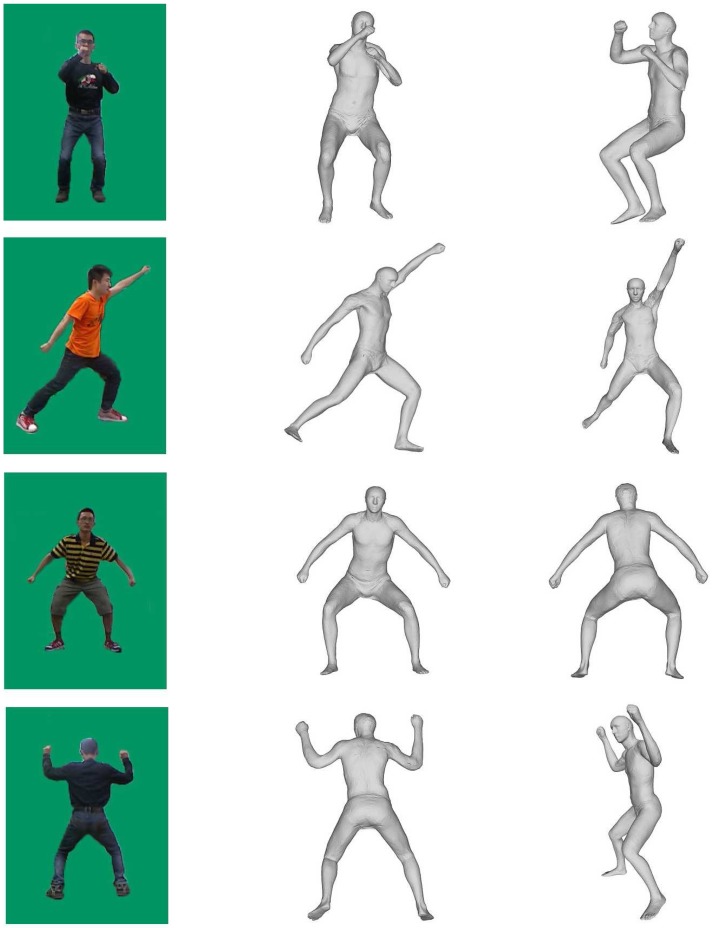
Results of the motion capture: Column 1 shows the RGB images from the left camera (for brevity, we do not show the RGB images from the right camera). Column 2 shows our results in the front view. Column 3 shows the results from viewpoints different from Column 1.

In this section, we demonstrate the effectiveness of our system by comparing against alternative methods. We have tested our system on a wide variety of human actions with a total of 50 sequences of video consisting of a wide range of human movements, including crossing arms, walking with a 360-degree rotation, standing up, playing baseball, and so on. There are 23 men and seven women, whose weights range from 42 kg to 93 kg and heights range from 1.55 m to 1.85 m, tested in our experiments (some people were tested more than once). Figure 5 shows some of our reconstructed poses. To reflect the advantages brought by the two Kinect sensors, we firstly compare our results against the skeletons from Kinect [20], which indicates that our solution deals with occlusions effectively. Then, further comparison against the motion capture using a single depth camera [15] proves that our method has a great improvement for the accuracy of pose estimation. We also validate the quality of our two-Kinect system by computing the average 3D joint position discrepancy between estimated poses and that from the ground truth, where the estimated poses include reconstructed poses from our two-Kinect system, Kinect SDK and Xu’s monocular system [15]. At last, the complexity and robustness of our system are evaluated.

### 4.1. Comparison against Kinect

**Figure 6:**
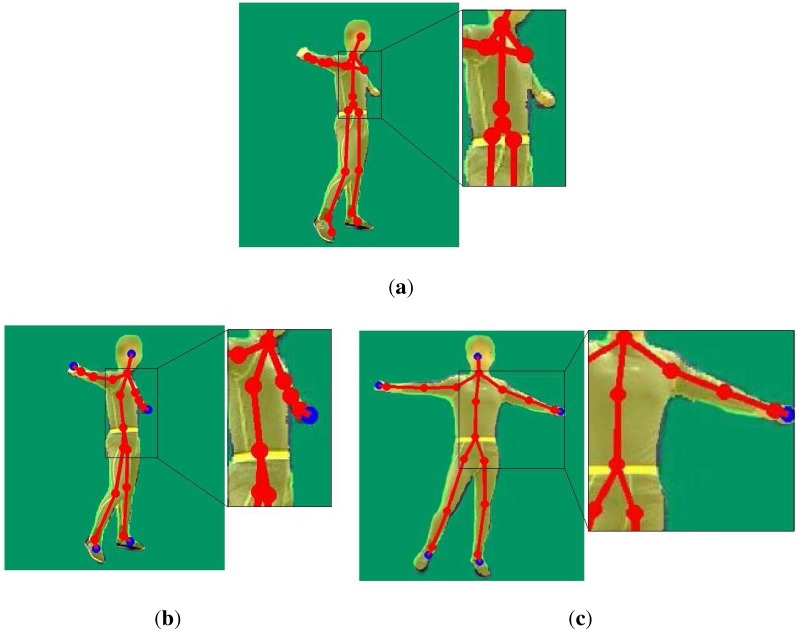
Comparison against Kinect: (**a**) skeletons from Kinect; (**b**,**c**) skeletons of our reconstructed model projections to the RGB images from the left camera and the right camera; the red dots are the joints of the model, and blue dots in (**b**,**c**) are the centers of the corresponding bones.

We compare our result against the skeletons from Kinect. We focus our comparison on capturing human movements in the presence of significant occlusion. Figure 6 shows the comparison. In Figure 6a, since some parts of the left arms are occluded by the human body, the Kinect gives a poor pose inference of the left arm, but our solution gives a good estimation, as shown in Figure 6b,c, as we can make full use of more depth data of the left arm from the right Kinect sensor, which prevents our estimation from having a poor inference. Furthermore, we can see that our reconstructed model is more accurate in the pose of the right arm and legs, as well as the location of the spine. In Figure 6a, the projection location of the spine is lower than the position of the spine in our model. In summary, the Kinect often gives a poor inference of the poses when significant occlusion occurs. Our two-Kinect sensor system can help solve this problem, as we can get more information of the movements from two viewpoints, so that we can use both depth maps as a further constraint. Obviously, the more depth cameras we use, the more accurate our pose estimation will be. However, the interference among these IR cameras may be a great challenge.

### 4.2. Comparison against Motion Capture Using a Single Depth Camera

**Figure 7:**
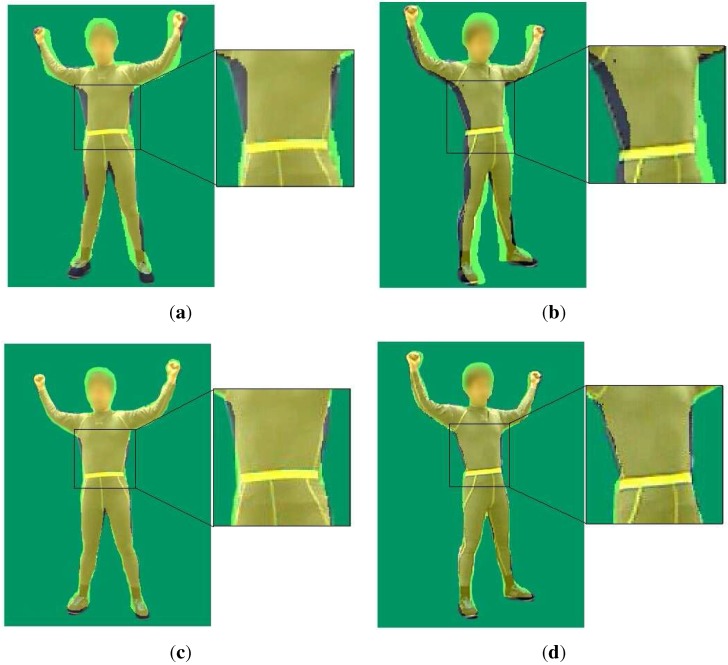
Comparison against [15]: (**a**,**b**) the reconstructed SCAPE model’s projection respectively using the depth data from the left and the right camera with the method of [15]; (**c**,**d**) our reconstructed model’s projection using the depth data from the left and the right camera at the same time.

**Figure 8:**
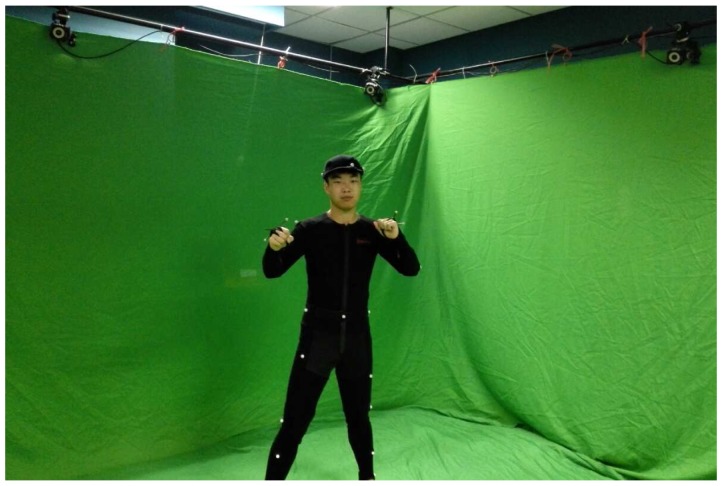
OptiTrack system setup for ground truth data.

We compare our results with the results reached in Xu *et al.*’s work [15]. We compare the results of these two methods by projecting our reconstructed model onto the RGB images and calculating the average proportion of the effective region. Figure 7 shows the qualitative comparison. As illustrated in Figure 7, our method gives a better estimation of the waist and chest in Figure 7c,d than that in Figure 7a,b. Comparing Figure 7b to Figure 7d, estimation of the legs by our method is more accurate than that by the method of [15]. The projection of the reconstructed model of [15] wanders further from the origin position of the human body than that of our method. The projection difference of the waists of the reconstructed poses is especially obvious in Figure 8.

**Figure 9:**
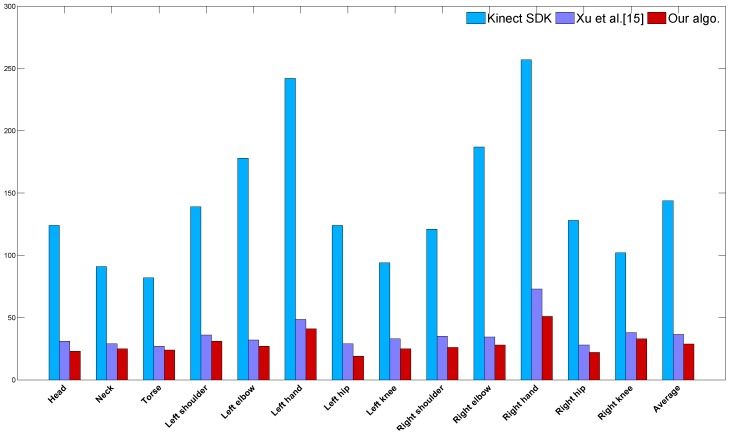
Quantitative comparison of the average reconstruction error. The vertical axis represents the error of joint position discrepancy, and its coordinate unit is mm. The horizontal axis successively shows the name of all of the joints, except that Column 14 shows the average error.

We compute the average 3D joint position discrepancy between the estimated poses and the ground truth motion capture poses, where the estimated poses consist of poses from the Kinect, poses derived from Xu’s method [15] and poses from our two-Kinect system. Figure 9 shows the quantitative comparison of the average reconstruction error.

We also have a quantitative comparison of these two methods, as shown in Table 2: our solution has a great improvement in the proportion of the effective region, which results in an increase of about three percentage points in the average coincidence ration of the effective area. Still, the proportions of the effective region are different from the viewpoints of the left and right cameras, which is caused by the different distance away from the two cameras and the different depth accuracy. Additionally, the proportion of the effective region of our method in Table 2 is different from that in Table 1, as we use more poses to evaluate the average proportion in Table 2. Moreover, we find that the pose tracking of [15] fails about every 25 frames, while our pose tracking fails about every 40 frames, which shows that our system is more robust.

**Table 2:** Quantitative comparison of our pose tracking with [15]. The effective region means the region where the pixels belong to both the observed and the rendered map and the difference of the depth value is less than 5 cm.

	Left Camera	Right Camera
Proportion of the effective region of [15]	86.35%	87.73%
Proportion of the effective region of our method	89.72%	90.86%

### 4.3. Comparison against OptiTrack

We quantitatively assess the quality of our system by comparing against data from the ground truth. The ground truth data are from our eight-camera OptiTrack system with a full marker set, whose setup is shown in Figure 8.

As illustrated in Figure 9, the average reconstruction error of our method is about 2.9 cm per joint per frame, while that is 14 cm for Kinect SDK and 4 cm for Xu’s method. This comparison result shows that our method gives a more accurate estimation for human motion.

**Figure 10:**
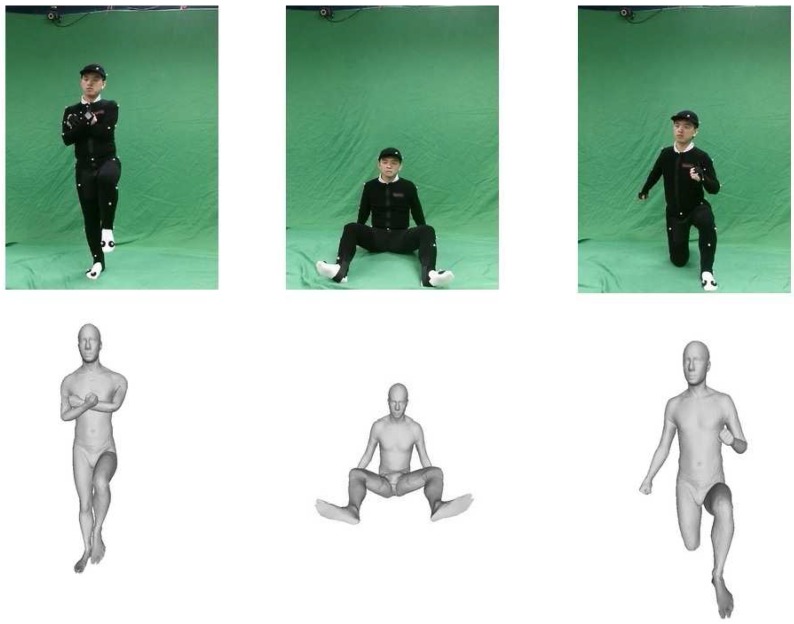
Difficult poses. Figures on the top are RGB images from the left camera. Figures on the bottom are reconstructed models from the front view.

**Figure 11:**
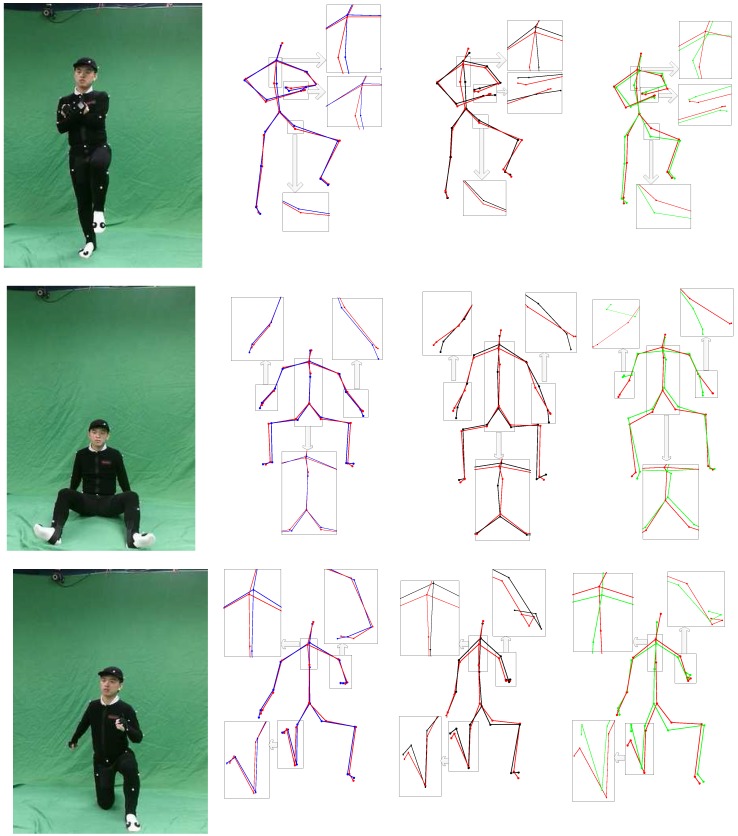
Skeletons of difficult poses obtained by the OptiTrack system, Xu’s monocular setup [15], the Kinect SDK [20] and our two-Kinect system. The first column shows the RGB images from the left camera. Column 2 shows a comparison of our reconstructed poses and that from the OptiTrack system, where the red skeletons are from the OptiTrack system and the blue are our reconstructed results. Red skeletons in Column 3 are also from the OptiTrack system, and the black ones are derived by Xu’s method [15]. In Column 4, the green skeletons are obtained from the Kinect SDK, while the red are from the OptiTrack system.

Furthermore, we conduct a detailed analysis on some difficult poses, which are suffering from significant occlusions, as shown in Figure 10 and Figure 11. Figure 10 shows these three difficult poses by the RGB images from the Kinect RGB sensors and their reconstructed models from the front view. Figure 11 illustrates the skeletons obtained by the OptiTrack system, Xu’s monocular setup [15], the Kinect SDK [20], as well as our two-Kinect system.

It is visually demonstrated that our two-Kinect system can produce good estimations of difficult poses with significant occlusions in Figure 10. From Figure 11, we can see that the Kinect often gives a poor inference of occluded body parts, and joints often deviate from the ground truth data more than our two-Kinect system. As our two-Kinect system could not acquire all of the point cloud data of a model, we may give estimations with a higher error for some bodies, which are occluded from both viewpoints of our two Kinect cameras, seen as the righnet arm in the second row and the right leg in third row in Figure 11. However, our system often gives better estimations of these parts than Xu’s method [15], as our temporal term gives a good prediction of these parts.

### 4.4. Evaluation of Algorithm Complexity and System Robustness 

#### 4.4.1. Complexity Analysis

Our system is conducted on the platform of MATLAB 2013 run on a computer whose CPU is the Intel Core i7-3770 at 3.4 GHz with RAM of 16 GB. The average time for motion capture is about 9.84 s per frame, while it is about 6.32 s per frame for Xu’s method [15]. It costs about 33 ms per frame for the Kinect SDK to capture the human pose. Although the Kinect SDK costs less time, it cannot reach the accuracy of our method. In our optimizations, the most time-consuming part is to use the the technology of kd-tree to find correspondences, which include that between the projection of the current template and the depth map, the silhouette map, as well as the RGB image. It is known that the complexity of kd-tree is $O\left(n\log n\right)$. Therefore, the complexity of our algorithm is $O\left(n^{2}\log n\right)$, as we should find correspondences of all effective points in our template (*n* represents the number of all effective points in the template), where the effective points indicate points that can be directly projected to the camera plane. Our complexity is the same as Xu’s monocular system, and our time cost of capturing one frame is theoretically at least two times that of Xu’s system, because we get the depth and RGB images from two Kinect sensors at the same time and we add a temporal term. However, our time cost of capturing one frame is even shorter than two-times that of Xu’s method, because more comprehensive movement data bring a more accurate estimation, which provides us the initialization of each frame closer to the current pose.

#### 4.4.2. Tracking Failure Analysis

For each tested sequence, we compute the percentage of the total frames that were switched to the pose detection module. On average, pose tracking of [15] fails about every 25 frames, while our pose tracking fails about every 40 frames. This means that 2.44% of the total frames in our system was re-initialized by the pose detection module, while the rest of the frames was automatically reconstructed by the pose tracking module. Additionally, 3.84% of the total frames at least was re-initialized by the pose detection module if we just use the monocular Kinect camera for motion capture, like [15]. Comparing the failure modes in our two-Kinect sensor system and the monocular system, we find that both of these tracking systems often get stuck in local minima and often produce poor estimation when tracking extremely fast movement due to poor initialization of the tracking poses. Furthermore, the monocular system is more prone to give poor estimation when occlusion occurs, while the two-Kinect sensor system often gives more depth measurement from the other viewpoint, so that the pose tracking module infers less cloud points of the occlusion. Therefore, more movement data are provided by our two-Kinect sensor system, making our pose tracking more accurate and more robust.

## 5. Conclusions

In this paper, we developed an automatic motion capture system using depth data and RGB data captured by two Kinect sensors. The system is low cost and convenient to setup. The key contribution of our paper is using two Kinect sensors from two different viewpoints to capture human movements and getting more information about human motion, which is especially useful when significant occlusion occurs. Another contribution of our paper is extending a motion capture framework by utilizing the temporal information for pose estimation. Experiments show that our solution for estimating the pose parameters of the human body is more accurate than the present methods.
